# Molecular Dynamic Simulation of Collision-Induced Third-Body Formation in Hydrogen-Free Diamond-Like Carbon Asperities

**DOI:** 10.1007/s11249-016-0712-9

**Published:** 2016-07-08

**Authors:** Julian von Lautz, Lars Pastewka, Peter Gumbsch, Michael Moseler

**Affiliations:** Fraunhofer IWM, MicroTribology Center, Wöhlerstraße 11, 79108 Freiburg, Germany; Institute for Applied Materials, Karlsruhe Institute of Technology, Kaiserstraße 12, 76131 Karlsruhe, Germany; Institute of Physics, University of Freiburg, Herrmann-Herder-Str. 3, 79104 Freiburg, Germany

**Keywords:** Tribo-induced phase transformation, Rehybridization, Third body, Diamond-like carbon, Asperity collision, Shear bands, Geometric overlap model

## Abstract

The collision of two cylindrical hydrogen-free diamond-like carbon (DLC) asperities with approximately 60 % sp^3^ hybridization has been studied using classical molecular dynamics. The severity of the collision can be controlled by the impact parameter *b* that measures the width of the projected overlap of the two cylinders. For a cylinder radius of *R* = 23 nm, three collisions with *b* = 0.5 nm, *b* = 1 nm and *b* = 2.0 nm are compared. While for the two small *b* a single shear band between the collision partners and a strongly localized sp^2^/sp^1^ hybridised third-body zone between the asperities is observed, the *b* = 2 nm collision is accompanied by pronounced plastic deformation in both asperities that destabilize the metastable sp^3^-rich phase leading to a drastic increase in the amount of rehybridized tribomaterial. In addition, pronounced roughening of the cylinder surfaces, asymmetric material transfer and the generation of wear debris are found in this case. For the *b* = 0.5 and 1 nm collision, the evolution of third-body volume can be quantitatively described by a simple geometric overlap model that assumes a sliding-induced phase transformation localized between both asperities. For *b* = 2 nm, this model underestimates the third-body volume by more than 150 % indicating that plasticity has to be taken into account in simple geometric models of severe DLC/DLC asperity collisions.

## Introduction

The tribological behavior of technically relevant systems is closely related to sliding-induced phase transformations [1] that generate a so-called third body [2–4] between two counter surfaces. Usually, third bodies form during running-in [5] and the fate of the tribological system is determined by the properties of the third-body material [6]. In general, a successful running-in results in a well-formed third-body and a long-lived tribosystem with low friction and wear, while a system with a third body that is not stable results in early failure [7]. It is therefore essential to understand the microscopic mechanisms that govern the formation of third-body layers as well as their properties such as structural composition and thickness.

Metallic materials are used in most technical applications. In this case, the running-in processes are rather complicated. Experimental [1, 8, 9] and first theoretical [4, 10] studies indicate that an interplay between a complex tribochemistry (involving many elements from the base materials and the lubricants) and crystal plasticity [11–13] result in quite heterogeneous third bodies that gradually evolve from the microcrystalline base material, via a grain-refined zone [7, 9] into an amorphous top layer [14]. Consequently, the detailed microscopic understanding of the underlying mechanism progresses rather slowly. Therefore, simpler yet technologically relevant systems, such as diamond [15–18], silicon [19] or carbides [10], are of significant interest.

Tetrahedral amorphous carbon (ta-C) is a hydrogen-free DLC [20]. Since it consists of mainly sp^3^-bonded amorphous carbon, chemical complexity is strongly reduced and crystal plasticity is absent. Therefore, ta-C represents an important model tribomaterial [21]. At the same time, ta-C is also a technically relevant coating [22], since it is used in an increasing number of tribological applications, ranging from hard disks [23] to diesel injection valves [24]. Most of ta-C’s beneficial properties derive from its diamond-like hardness that is related to a predominance of sp^3^ bonding among the carbon atoms [25].

Amazingly, even such a simple material can form a third body. Using energy-filtered transmission electron microscopy, Jolly-Pottuz et al. [26] observed the formation of a (predominantly sp^1^ and sp^2^ hybridized) nanoscale amorphous carbon (a-C) surface layer on top of a ta-C coating after sliding (very similar to the a-C tribolayer found on polished diamond surfaces [17, 18]). This experimental observation was later on verified by Kunze et al. [21] employing atomistic simulations. In Kunze’s study, the contact of two completely flat ta-C surfaces (with 70 % sp^3^ content) was simulated by classical molecular dynamics. A shear band was observed in the a-C layer that formed during sliding. It was noticed that the plasticity in the shear band triggered the hybridization in the neighboring bulk sp^3^ material leading to a slow vertical growth of the a-C layer with increasing sliding distance.

**Fig. 1:**
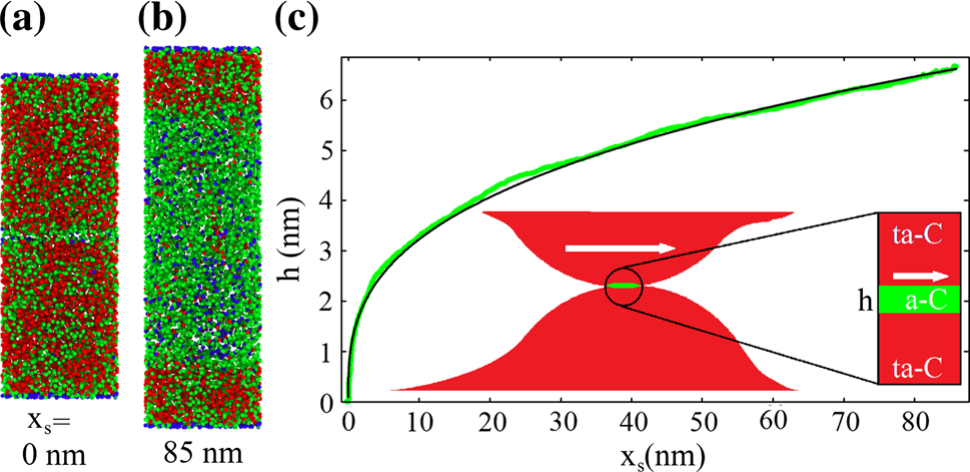
Evolution of an a-C layer (*green zone*) that forms between two flat ta-C surfaces with initially 60 % sp^3^ hybridization during an MD simulation with a reactive bond-order potential (as describe later on in the methods section). **a** Snapshot of the initial system (size 2.9 × 2.9 × 8.2 nm^3^). *Blue*, *green* and *red* spheres represent carbon atoms in sp^1^, sp^2^ and sp^3^ hybridization state, respectively. **b** Snapshot after sliding the upper ta-C block for a total sliding distance of *x*_*s*_ = 85 nm with a velocity of *v* = 10 m/s. **c** Height *h* of the a-C layer (*green zone* in **b**) that forms during sliding (*thick green line*). *h* is determined by fitting a Fermi function to the sp^3^ evolution at the ta-C/a-C interface. The locations of the dividing surfaces between a-C and ta-C are defined by the heights where sp^3^ has dropped to 40 %. The black line represents a power law fit to the MD data. The *inset* in **c** illustrates the approximation of an asperity collision by such a periodic calculation with two flat counter bodies

Figure 1 shows the result of an MD simulation similar to Kunze’s original work [21]. The initial system (panel a) consists of two ta-C counter bodies (reddish zones with 60 % sp^3^ hybridization) separated by a small layer of a-C (greenish zone). After sliding the upper counter body for a total sliding distance of *x*_*s*_ = 85 nm, an a-C layer with a height *h* = 8.6 nm has formed (panel b). The width *h* of the sp^2^-rich a-C zone as a function of the sliding distance *x*_*s*_ shows at the beginning of the simulations a fast increase and continues to grow later on with a smaller growth rate [21] (see, for instance, the green curve in panel c of Fig. 1). The evolution of *h* can be quantitatively described by a power law

1 $$h(x_{s} ) = Ax_{s}^{1/3}$$

(see black curve in panel c of Fig. 1). Although such a power law can be microscopically motivated for the a-C formation on diamond surfaces [15], for ta-C the nature of Eq. (1) is still purely empirical. The empirical prefactor *A* determines the growth rate of the a-C and can be extracted from the MD simulations. It depends on the ta-C’s initial sp^3^ fraction. For instance, the evolution of *h* shown in Fig. 1c for a ta-C with 60 % sp^3^ can be described by $A \approx 1.5$ nm^2/3^.

In principle, such an empirical local constitutive equation in combination with a plasticity model for the a-C could be used in continuum simulations [27] to describe third-body formation and topography evolution in macroscopic ta-C pairings. However, it is not immediately evident that the simple geometry considered in Ref. [21] is sufficient to determine a quantitative local constitutive equation for third-body formation in a multiscale approach.

The reason why MD simulations of two flat ta-C surfaces potentially provide useful information for tribologically loaded technical ta-C coatings is illustrated in the inset of Fig. 1c. Although some ultrathin ta-C coatings are reported to be ultrasmooth [28], most ta-C surfaces exhibit a certain roughness [29], and therefore, only a small fraction of apparent surface area is in contact [30]. The real contact area is formed by the highest asperities of both surfaces [31]. During continuous sliding, these asperities collide (as indicated by the inset in Fig. 1c) and contacts are formed and broken within a timescale of ns to µs for sliding speeds of the order of 1 m/s.

Locally, the collision of two flat asperities resembles two flat surfaces in sliding contact (inset of Fig. 1) provided the collision is mild enough to accommodate the induced shear strain in a region close to the interfaces. Therefore, understanding a-C formation between two flat ta-C surfaces is likely to be a first step toward a description of the corresponding tribologically induced phase transformation on rough extended surfaces.

In this article, it will be explicitly tested whether the collision of two ta-C asperities can be reduced to the local description outlined in Fig. 1c. This will be accomplished by comparing the third-body evolution in large-scale atomistic calculations of ta-C asperity collisions with the outcome of a geometric overlap model (GOM) that employs Eq. (1).

## Methods

The collision of two cylindrical asperities is studied by classical molecular dynamics employing Brenner’s second-generation reactive bond-order potential (REBO2) [32]. In REBO2, the total energy as a function of the atomic positions is given by a sum over repulsive (*V*^R^(*r*_*ij*_)) and attractive contributions (*b*_*ij*_*V*^A^(*r*_*ij*_)) of individual bonds (with length *r*_*ij*_ and a bond order *b*_*ij*_)

$$U(\vec{r}_{1} , \ldots ,\vec{r}_{N} ) = \sum\limits_{i < j} \left[ {V^{\text{R}} (r_{ij} } ) - b_{ij} V^{\text{A}} (r_{ij} ) \right]$$

The sum in this expression involves first nearest neighbor interactions only and parameters in the functions *V*^R^(*r*_*ij*_), *V*^A^(*r*_*ij*_) and *b*_*ij*_ have been fitted to a variety of carbon and hydrocarbon systems. Nearest neighbors are determined by a distance-dependent cutoff scheme. In this form, REBO2 has been used in numerous atomistic studies—even for phase transformations [33] in ta-C. However, distance-dependent cutoff schemes result in a severe overestimate of the force to break C–C bonds. A correct description of plasticity, fracture and the density in amorphous carbon phases requires a more sophisticated first nearest neighbors definition [34–36] determined by a screening method [34].

**Fig. 2:**
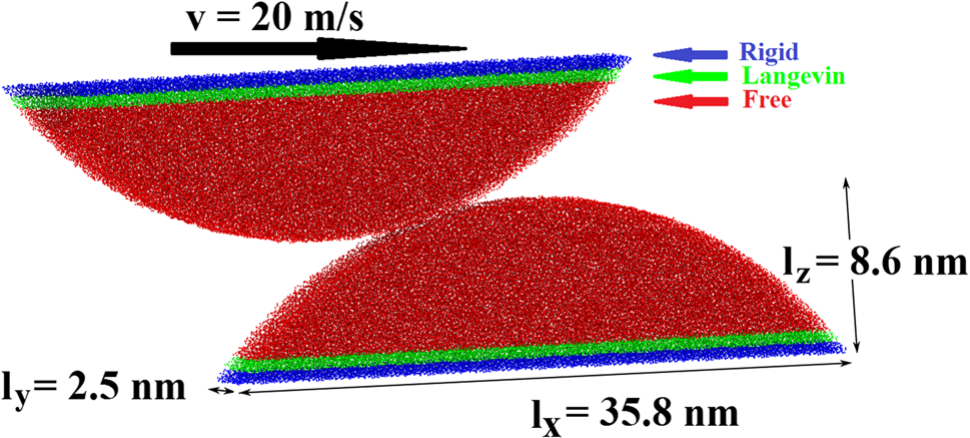
Setup of the atomistic simulation: *blue* atoms are kept rigid, *green* atoms are thermalized using a Langevin thermostat, and *red* atoms are free to move. The system employs periodic boundary conditions in the *y*-direction. A slightly tilted system is presented in order to expose also the third dimension of the system with a periodic repeat length *l*_*y*_ = 2.5 nm

Both cylindrical asperities consist of 82,257 carbon atoms and have a cap length of *l*_*x*_ = 35.8 nm, a height of *l*_*z*_ = 8.6 nm and a periodic repeat length of *l*_*y*_ = 2.5 nm (Fig. 2). Each asperity is cut from a rectangular ta-C block (with 60 % sp^3^ hybridization), deleting all atoms outside an *R* = 23 nm radius cylinder.

**Fig. 3:**
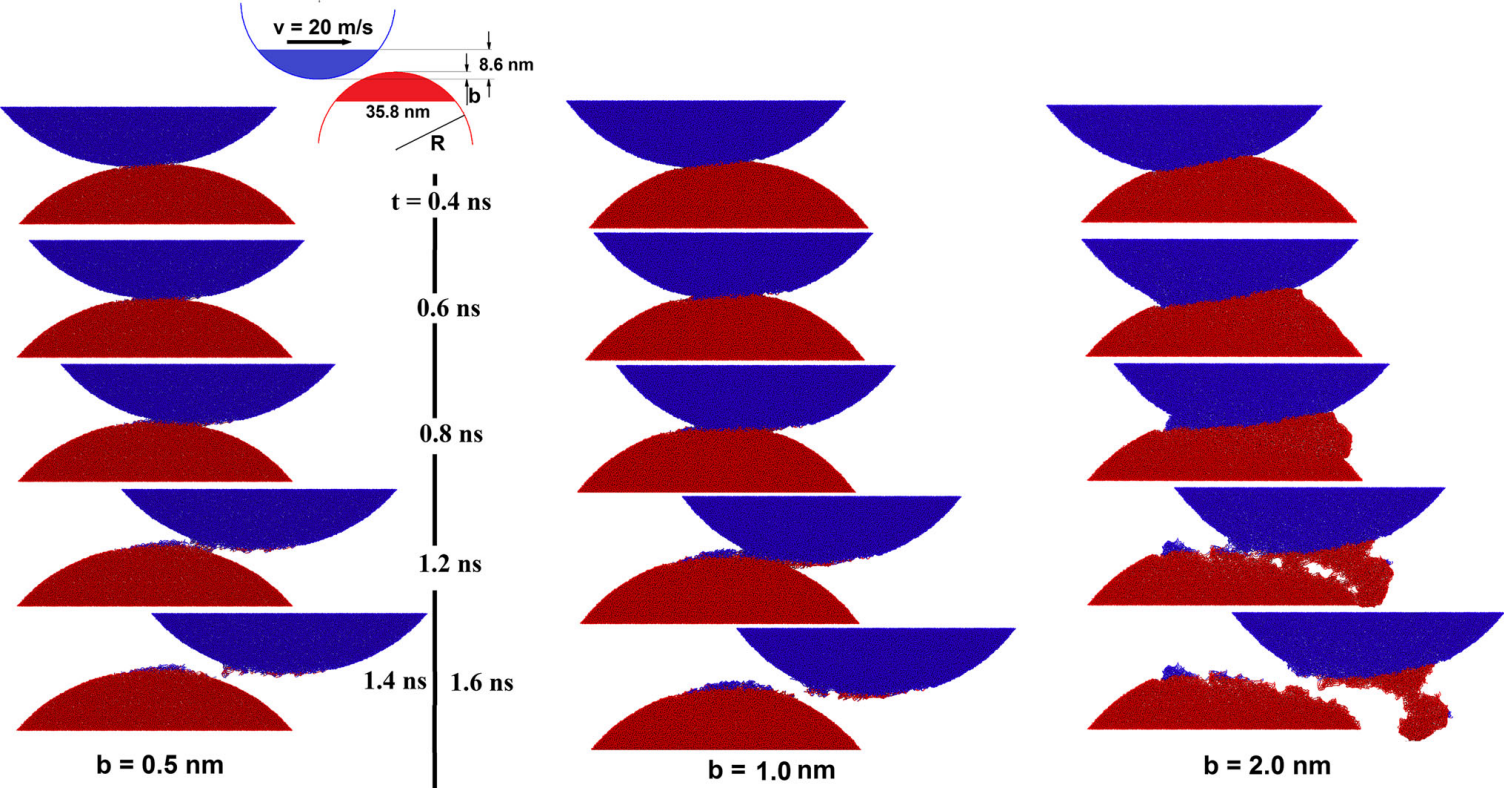
Snapshots of the dynamics of three ta-C asperity collisions with different impact parameters *b*. Atoms in the upper asperity are displayed in *blue* and atoms in the lower asperity in *red*

The width of the overlap of both asperities (projected on the plane normal to the sliding direction) can be considered a control parameter describing the severity of such a collision. In the following, we call this quantity the impact parameter *b* (see sketch in Fig. 3). Ideally, simulations with various cylinder radii and a large range of impact parameters should be performed for a complete and generalizable estimate of the influence of topography on third-body formation on ta-C. Unfortunately, computer time requirements of molecular dynamics simulations with screened bond-order potentials are too demanding for exhaustive parameter variations. In this article, a first exploratory contribution will be given by considering one cylinder radius (*R* = 23 nm) and three different impact parameters *b* = 0.5, 1 and 2 nm.

In the lower (upper) asperity, the bottommost (upmost) atoms in a 0.6-nm-thick layer are treated as a rigid body (marked blue in Fig. 2). Another 0.6-nm layer of atoms in the neighborhood of both rigid zones are thermalized in the direction perpendicular to sliding at 300 K during the whole simulation by Langevin dynamics [37] employing a Langevin dissipation constant of 1 ps. Contact of the asperities is achieved by constantly displacing the block of rigid atoms in the top asperity at a velocity of *v* = 20 m/s in x-direction. The equations of motion for all non-rigid atoms are integrated by the velocity Verlet algorithm with a time step of 0.5 fs [38].

For all three *b*, no passivation of the asperities (for instance by hydrogen) was considered, since the main focus of this article rests on the plasticity and phase transformations triggered by their cold welding. During the simulation, the coordination (hybridization) of every atom is calculated, by counting the number of neighbors inside a cutoff radius of 1.85 Å (the minimum between first and second nearest neighbor distances in the C–C radial distribution of the ta-C).

Local strain is computed by determining the minimal deformation gradient tensor that maps atomic positions between a local reference configuration and the current local configuration [39] within a radius *r*_L_ = 5 Å. This radius represents a compromise between a high local resolution (small *r*_L_) and stable statistical averages (large *r*_L_). For the chosen *r*_L_, local reference configurations consist of approximately 100 carbon atoms.

From the MD trajectories, the total a-C third-body volume is calculated as follows. The cross-sectional area of both cylindrical caps is covered by a rectangular grid resulting in bins of approximate size 0.75 × 0.75 nm^2^. The sp^3^ fraction in each bin is calculated as the ration of fourfold-coordinated C atoms to the total number of C atoms in the bin. A bin is considered filled with a-C when the sp^3^ fraction has dropped below 40 %. This value is half-way between the initial sp^3^ (60 %) and the final sp^3^ (10–20 %) of the fully transformed layer. The total a-C volume of both asperities *V*_a-C_ is computed as the number of a-C filled bins times bin area times periodic length l_y_.

## Results of the MD Simulations

Figure 3 displays snapshots of the three collisions. Atoms are color-coded according to their initial position (blue: in the upper asperity, red: in the lower one). We start with a description of the *b* = 0.5 nm collision. The line contact that forms immediately after the asperities touch evolves quickly into a cold-welded area that connects both asperities by covalent bonds (snapshot at *t* = 0.4 ns). This area moves to the right during further sliding of the upper asperity (*t* = 0.6 and 0.8 ns) and is accompanied by the formation of an atomically thin transfer film on the lower asperity (see blue atoms on the surface of the red asperity at *t* = 0.8 ns). At *t* = 1.2 ns, both asperities start to separate. Transferred atoms are now visible on both asperities.

The snapshot at *t* = 1.4 ns shows both asperities after complete separation. The asperities deviate only slightly from their cylindrical shape. Both surfaces are covered with sp^1^ carbon chains that originate from the upper and lower tribopartner.

The structural evolution in the *b* = 1 nm case follows a similar pattern as in the *b* = 0.5 nm case (compare left and middle column in Fig. 3) with a more pronounced transfer film formation.

The scenario is different for the *b* = 2 nm collision. The cold-welded area that forms at the beginning (*t* = 0.4 ns) later on broadens (*t* = 0.6 and 0.8 ns) and becomes comparable in dimension to the lateral size of the asperities. This process is accompanied by a localized plastic deformation on the lower asperity (*t* = 0.6 ns). The strongly deformed amorphous carbon piles up at the right side of the bottom asperity.

Note that the localized deformation does not occur on the upper asperity. In this asperity, a more uniform deformation is apparent, clearly breaking the symmetry of the collision dynamics at around at *t* = 0.8 ns. Subsequently, local bulging out of carbon occurs also on the left side of the upper asperity; however, bulge formation on the right side of the lower asperity is significantly more pronounced. In contrast to previous work carried out under more severe conditions [12], no intermixing of the asperities during the collision can be detected.

At *t* = 1.2 ns, separation of the asperities has started. The large cold-welded area is now under tension which is relieved by pore formation in the bulk of the contact. Between these pores, several bridges (of various size) still maintain the contact between both tribopartners. As already observed during the milder collisions (*b* = 0.5 and 1 nm), the smallest bridges consist of linear carbon chains. Such chains have already been reported during the separation of cold-welded flat amorphous carbon surfaces [40].

Finally, the pores coalesce into a large void that mainly passes through regions initially belonging to the lower asperity. Hence, material transfer to the upper asperity can be observed after the final detachment (see large red region at *t* = 1.6 ns). The surfaces display an atomically rough surface, forming valleys where the contact between the asperities was first broken. Interestingly, a particle has been generated that is merely connected to the upper asperity by a thin carbon bridge. Since the complete detachment of this particle during a subsequent collision is likely, this process indicates one possible mechanism to explain wear in ta-C. Note that another wear mechanism is based on chemical etching of the carbon chains by oxygen molecules [40, 41].

**Fig. 4:**
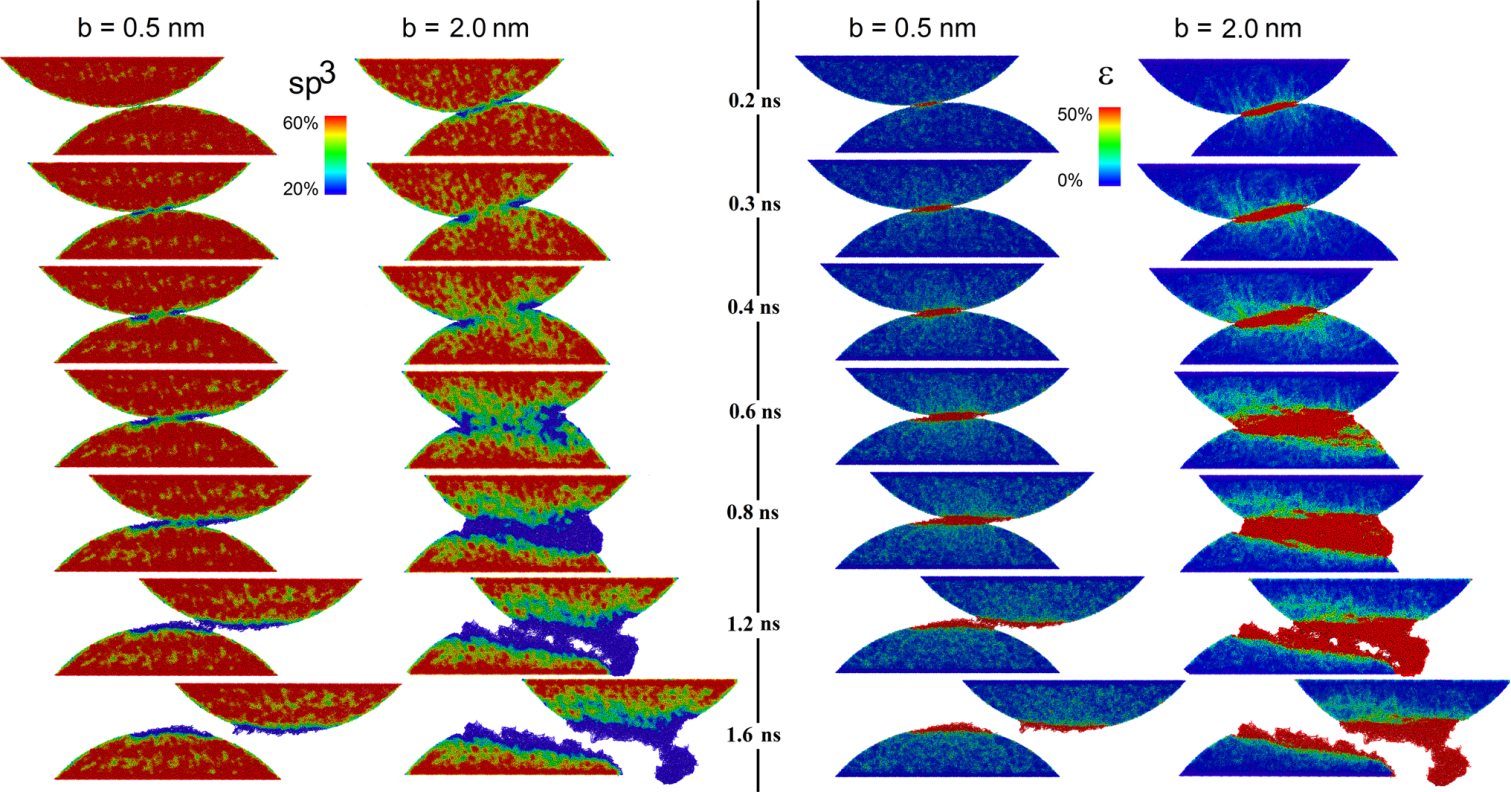
Evolution of local hybridization (*left two columns*) and local von Mises strain (*right two columns*) during the collision of two ta-C asperities

In the following, we address one central question of this article, namely the dependence of the evolution of third body on the impact parameter *b*. Snapshots of the spatial distribution of the sp^3^ hybridization during the *b* = 0.5 nm and *b* = 2 nm collision are displayed in the first and second column in Fig. 4, respectively. The hybridization in the *b* = 1 nm collision is not displayed since it is similar to the *b* = 0.5 nm case. Starting with a description of the phase transformation in the *b* = 0.5 nm collision, one should note that already before the collision both asperities are covered with an atomically thin sp^1^- and sp^2^-rich a-C layer (green zones in Fig. 4 at *t* = 0.2 ns) as also found in several experimental studies [26, 42, 43]. Immediately after contact formation (at *t* = 0.3 ns), the a-C layer between the asperities grows. Upon sliding, the contact zone (and consequently the a-C growth zone) moves on the asperity surfaces (to the right on the lower and to the left on the upper asperity) leaving behind fresh surfaces with pronounced sp^2^ character (blue regions in Fig. 4 at *t* = 0.4, 0.6 and 0.8 ns). After asperity separation (at *t* = 1.2 ns), both asperities are covered with an approximately 1-nm-thick a-C layer (blue crescents at *t* = 1.6 ns). Additional small patches with increased sp^2^ can be observed in the bulk of the asperities. These occur presumably due to their elastic loading. It has been argued by Kunze et al. [21] that even stresses below the yield stress of the material can break prestrained bonds outside the region where plastic deformation is localized. The amount of rehybridization at small strain and stress likely depends on the age of the a-C.

**Fig. 5:**
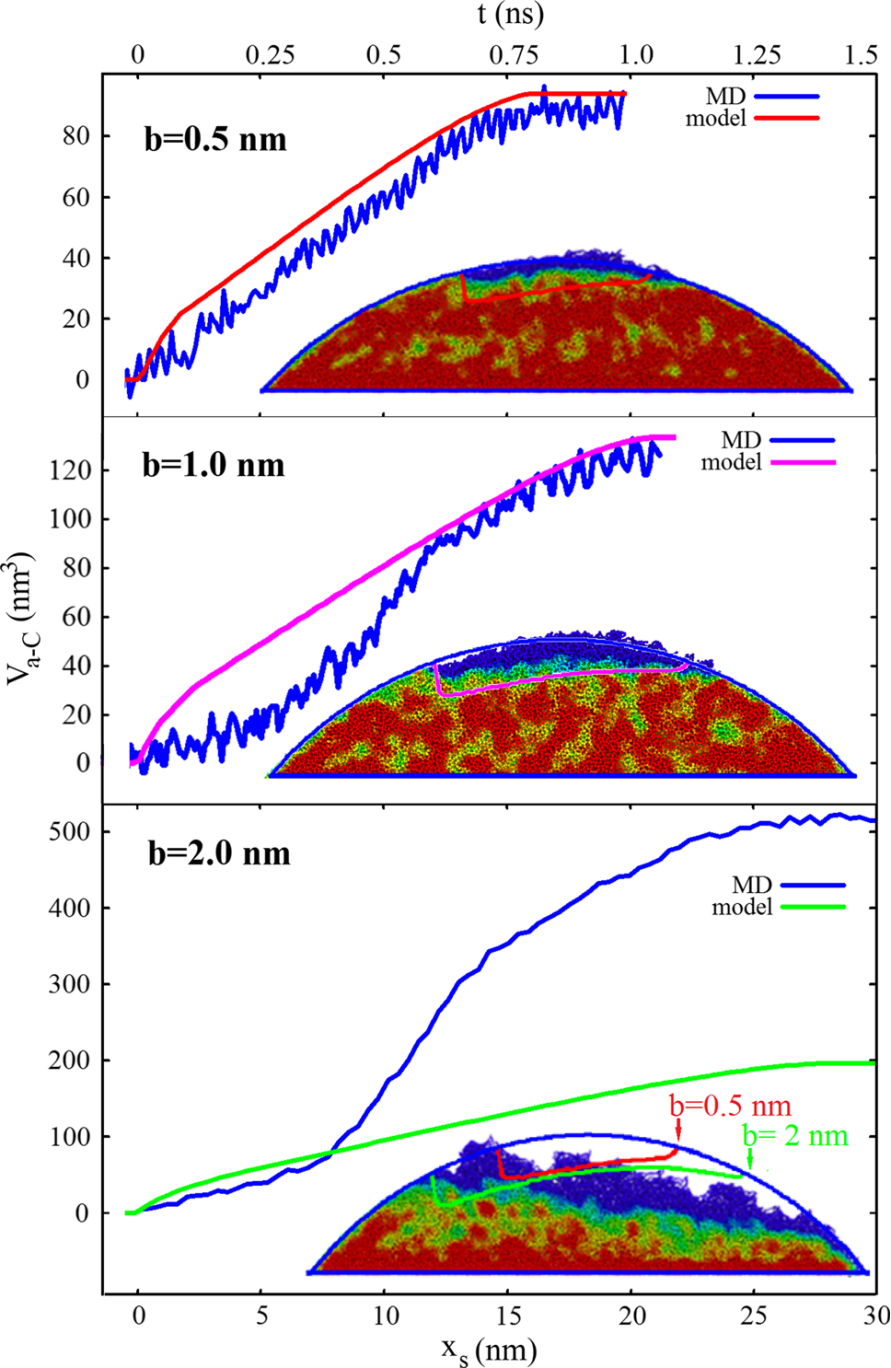
Evolution of the third-body volume *V*_*a*–*C*_ for the three asperity collisions *b* = 0.5, 1, 2 nm as a function of sliding distance *x*_*s*_ of the upper asperity. The asperities establish full contact at *x*_*s*_ = 0 nm. The top abscissa shows time (according to *t* = *x*_*s*_ */v*). The *blue curves* display the results from our classical MD simulations, while the *red*, *purple*, and *green curves* show the prediction of a simple geometric overlap model (GOM) that is based on the a-C growth behavior between two flat ta-C surfaces (Fig. 1). The *insets* compare the shape of the final a-C layer as obtained by our MD simulations with the prediction of the GOM

Figure 5 displays the total volume *V*_a-C_ of the a-C layer on both asperities as a function of sliding distance *x*_*s*_ of the upper asperity. Starting from the time when contact between the cylinders is established (*x*_*s*_ = 0 nm) until the cylinders separate again (*x*_*s*_ = 16 nm), a linear increase *V*_a-C_ can be observed (see blue curve in the top panel of Fig. 5). After the collision, almost 90 nm^3^ ta-C has formed. For the *b* = 1 nm collision, the evolution of the a-C volume follows a similar trend (although slightly deviating from linearity) with a final a-C volume of 120 nm^3^

In the *b* = 2 nm collision, third-body formation starts the same way as in the milder cases by the growth of an a-C surface layer (see second column in Fig. 4 at *t* = 0.2 and 0.3 ns). Soon, however, branches of the a-C region are formed that spread into the bulk of both asperities (green fingers at *t* = 0.4 ns). These branches grow, coalesce and form a broad a-C band between both tribopartners (blue/green zone at *t* = 0.6 and 0.8 ns). Asperity separation proceeds via void formation in the weakest part of this zone (the blue area at 1.2 ns) whereby the a-C is almost evenly divided between both asperities (see *t* = 1.6 ns). Interestingly, the particle that formed on the right-hand side of the upper asperity consists completely of a-C. Such “graphitic” wear debris is often found in wear experiments.

Initially, the volume of the a-C phase *V*_a-C_ grows linearly (see blue curve in bottom panel of Fig. 5). However, after a sliding distance *x*_*s*_ = 7 nm of the upper cylinder, plastic deformation sets in and the rate of a-C formation is drastically increased. After the collision, more than 500 nm^3^ a-C have been produced.

The observed rehybridization is intimately related to the evolution of the spatial distribution of plastic strain (third and fourth column in Fig. 4). Under milder conditions (*b* = 0.5 nm), the von Mises strain ε is strongly localized in the central region between both asperities. The evolution and location of this narrow shear band coincide almost exactly with the growth pattern of the corresponding a-C layer (first column in Fig. 3).

The shear band that forms at the beginning of the *b* = 2 nm collision (see ε in the fourth column of Fig. 4 at *t* = 0.2 and 0.3 ns) develops branches at later times (from *t* = 0.6 ns on). As for the *b* = 0.5 nm collisions, a significant correlation between this shear band and the location of the a-C zones can be seen.

## A Geometric Overlap Model

We can use the empirical Eq. (1) to derive a simple geometric overlap model (GOM) for the evolution of the a-C volume as a function of sliding distance *V*_a-C_(*x*_*s*_). Equation (1) is the solution of the ordinary differential equation

2 $$\frac{{{\text{d}}h(x_{s} )}}{{{\text{d}}x_{s} }} = \frac{{A^{3} }}{3}\frac{1}{{h(x_{s} )^{2} }} .$$

Since we aim at a description of a-C formation during the collisions of ta-C asperities with initially 60 % sp^3^, we use the parameter *A* = 1.5 nm^2/3^ from our independent simulation of two flat ta-C surfaces with 60 % sp^3^ displayed in Fig. 1. We now consider the collision of two cylinders of radius *R* = 23 nm with impact parameter *b*. The surfaces of the lower and upper cylinder are represented by a profile functions z_1_(*x*) and *z*_2_(*x*), respectively:

3 $$z_{1} (x) = \left\{ {\begin{array}{*{20}l} {\sqrt {R^{2} - x^{2} } } \hfill & {{\text{for}}\quad x| < R} \hfill \\ 0 \hfill & {{\text{for}}\quad x| \ge R} \hfill \\ \end{array} } \right.$$

4 $$z_{2} (x) = 2R - b - z_{1} (x - \sqrt {4Rb - b^{2} } ) .$$

In the following derivation, the upper cylinder is assumed to move with a velocity *v* to the right, i.e., the time-dependent profile *z*_2_(*x*−*vt*) = *z*_2_(*x*−*x*_*s*_) is considered. Here *x*_*s*_ = *vt* denotes the (time-dependent) sliding distance of the upper cylinder.

Within a geometric overlap model, contact is established for all *x* with *z*_1_(*x*) > *z*_2_(*x*−*x*_*s*_), and in this case, it is assumed that on both asperities an a-C layer grows symmetrically according to Eq. (2). If we denote the width of the a-C layer on the lower asperity by *h*_1_(*x, x*_*s*_), the growth of the lower ta-C layer can be described by

5 $$\frac{{{\text{d}}h_{1} (x,x_{s} )}}{{{\text{d}}x_{s} }} = \frac{{A^{3} }}{6}\frac{1}{{[h_{1} (x,x_{s} ) + h_{2} (x - x_{s} ,x_{s} )]^{2} }} .$$

Since a symmetric a-C growth is assumed for each contact point *x* with *z*_1_(*x*) > *z*_2_(*x*−*x*_*s*_), the a-C on the upper asperity evolves according to

6 $$\frac{{{\text{d}}h_{2} (x - x_{s} ,x_{s} )}}{{{\text{d}}x_{s} }} = \frac{{{\text{d}}h_{1} (x,x_{s} )}}{{{\text{d}}x_{s} }} .$$

Explicit integration of Eqs. (5) and (6) is straightforward. The *x* dimension is discretized in steps of Δ*x* = 0.25 nm, and for all *x* with *z*_1_(*x*) > *z*_2_(*x*−*x*_*s*_), the a-C height functions are updated according to

$$h_{1} (x,x_{s} + \Delta x) = h_{1} (x,x_{s} ) + \frac{{A^{3} }}{6}\frac{1}{{[h_{1} (x,x_{s} ) + h_{2} (x - x_{s} ,x_{s} )]^{2} }}$$

and

$$\begin{aligned} & h_{2} (x - x_{s} ,x_{s} + \Delta x) = \\ & h_{2} (x - x_{s} ,x_{s} ) + \frac{{A^{3} }}{6}\frac{1}{{[h_{1} (x,x_{s} ) + h_{2} (x - x_{s}^{{_{{}} }} ,x_{s} )]^{2} }}. \\ \end{aligned}$$

The volume of the a-C is given by the

7 $$V_{\text{a - C}} (x_{s} ) = l_{y} \int_{ - \infty }^{\infty } {[h_{1} (x,x_{s} ) + h_{2} (x,x_{s} )]{\text{d}}x} .$$

Here, *l*_*y*_ = 2.5 nm is the periodic length in the *y*-direction (see Fig. 2). The integral in Eq. (7) is approximated by a Riemann sum.

## Results of the Geometric Overlap Model

The top panel of Fig. 5 displays the evolution of *V*_a-C_(*x*_*s*_) for the *b* = 0.5 nm asperity collision. The curve for the GOM (red curve) follows closely the corresponding MD result (blue curve). Note that no free parameters were involved here. The inset in the top panel of Fig. 5 compares the hybridization in the final configuration of the *b* = 0.5 nm asperity collision with the prediction of the GOM. The red curve displays the boundary of the a-C layer: z_1_(*x*)–*h*_1_(*x*, *x*_*s*_ = ∞). It nicely follows the contour line that separates the ta-C from the a-C in the final MD configuration. Also for *b* = 1.0 nm, a reasonable agreement between the MD and the GOM is observed (compare purple with blue curve in the middle panel of Fig. 5).

For small sliding distances (*x*_*s*_ < 7 nm), the GOM shows also fair agreement with the MD results for the *b* = 2 nm case (compare green curve in bottom panel of Fig. 5 with the blue MD curve). However, as soon as (bulk) plasticity sets in, the a-C formation rate in the MD increases by a factor of 5–10 over the (approximately linear) a-C growth in the GOM. This leads to an underestimation of the final a-C volume by around 60 %.

While the shape of the final a-C zone for the two milder collisions agrees well between MD and GOM, the quantitative description of this shape by the GOM fails completely for *b* = 2 nm (see green curve in the inset to the top panel of Fig. 5). The GOM predicts a thicker a-C layer on the left-hand side of the asperity. In the final MD configuration, the ta-C/a-C-boundary falls off to the right. We believe that bulk plasticity, shear band formation and eventually fracture are the processes responsible for this behavior. These are obviously not captured by the simple approximation of the GOM.

Although it could be questioned whether the finite system size used in the MD simulations exaggerate the observed shear banding for *b* = 2 nm, thus increasing the observed a-C volume and at the same time spoiling the agreement between the MD and the GOM, it is unlikely that the qualitative differences between the *b* ≤ 1 nm and the *b* = 2 nm results are caused by a too small system size, because the maxima of the subsurface von Mises stress are within the MD zone in all three cases.

## Discussion and Conclusions

In this article, the collision of two cylindrical ta-C asperities is studied by classical molecular dynamics. Three different impact parameters are considered resulting in asperity collision that could be characterized as mild (smaller two impact parameters) and severe (largest impact parameters). In all three cases, a phase transformation from the 60 % sp^3^ ta-C to a 10–20 % sp^3^ a-C can be observed suggesting that an third body consisting of low-density a-C is generated during sliding of rough ta-C surfaces. This is in agreement with experimental observations [26].

Under mild collision conditions, the sliding-induced shear strain, as well as the formed a-C, is localized on the cylindrical surfaces of both asperities. This is in qualitative agreement to the formation of an a-C layer between two flat surfaces localized in a single slowly growing shear band. Here, it is demonstrated that a constitutive equation for the a-C evolution between flat surfaces can even be used to quantitatively predict the outcome of the mild collisions within a geometric overlap model (GOM).

However, the geometric overlap model fails for the *b* = 2 nm collision. In this case, plasticity is no longer localized in the contact region of the asperities. Branched shear bands extend into the bulk of the asperities and drive the formation of a third body in their interior. Indeed, the GOM suggests a distinction between mild and severe wear as the point where the GOM breaks down. This definition is in agreement with empirical conclusions drawn from a simple visual inspection of the MD trajectories shown in Fig. 3.

The intimate relationship between plasticity and a-C formation can be explained by the well-known metastability of the ta-C. The sp^1^- and sp^2^-rich a-C is energetically more favorable than the ta-C [20]. As pointed out by Kunze et al. [21], some of the bonds that connect sp^3^ carbon atoms to their next neighbors are strongly strained. Application of a small additional external strain results in breaking of such a bond and changes the coordination of the bond’s carbon atoms. This can be considered the elementary mechanism of the sp^3^–sp^2^ transformation in ta-C. Clearly, as soon as stress becomes high enough for the asperity to flow, many of these unstable bonds break and a-C formation is strongly enhanced. We note that the details of this process likely depend on fine details of the microstructure of the a-C itself that depends on its route of formation and its age. The GOM that only takes into account plasticity on the surface of the asperities naturally breaks down as soon as bulk plasticity in the asperities is triggered.

In addition to elucidating the a-C formation, the atomistic simulations of asperity collisions provide useful information regarding the wear mechanisms and topography evolution of rough ta-C surfaces. For the mild collisions that involve cold welding, only little changes of the cylindrical asperity shape is detected. During separation, a few linear carbon chains are produced. Since these chains are instable with respect to chemical etching by O_2_ [40, 41], mild collisions result in mild wear mediated by CO_2_ formation.

The *b* = 2 nm collision results in roughened cylinders with many carbon chains on top. Consequently, chemical etching is enhanced in severe collisions. In addition, the precursor of an a-C wear particle is detected. Since these particles are observed next to experimental wear tracks, one might speculate about them originating from collisions that involve strong plastic deformations inside the asperities.

Since a-C is a rather ductile material, one could have expected that necking would occur during asperity separation (as, for instance, seen in metals [44]). Interestingly, however, the separation evolves via pore formation and the development of a long crack indicating that also fracture mechanical concepts have to enter a general continuum growth model that describes third-body formation on ta-C.
